# Extension for Community Healthcare Outcomes (ECHO) Chronic Pain & Opioid Stewardship in Northwestern Ontario: A Thematic Analysis of Patient Cases

**DOI:** 10.1080/24740527.2022.2126754

**Published:** 2022-11-28

**Authors:** Patricia A. Poulin, Yaadwinder Shergill, Adrian Grebowicz, Inês Almeida, Rosemee Cantave, Bryan MacLeod, Tim Larocque, Donna Garstin, Sarah F. Fitzgerald, Joshua A. Rash

**Affiliations:** a St Joseph’s Care Group, Project ECHO Chronic Pain & Opioid Stewardship, Thunder Bay, ON, Canada; b Department of Psychology, Ottawa Hospital, Ottawa, ON, Canada; c Clinical Epidemiology Program, Ottawa Hospital Research Institute, Ottawa, ON, Canada; d Faculty of Medicine, Department of Anesthesiology and Pain Medicine, University of Ottawa, Ottawa, ON, Canada; e Department of Health Research Methods, Evidence, and Impact, McMaster University, Hamilton, ON, Canada; f Northern Ontario School of Medicine, Lakehead University, Thunder Bay, ON, Canada; g Department of Psychology, ISCTE – Instituto Universitário de Lisboa, Lisbon, Portugal; h St. Joseph’s Care Group, Chronic Pain Management Program, Thunder Bay, ON, Canada; i School of Social Sciences, Memorial University of Newfoundland, St. John’s, NL, Canada

**Keywords:** Chronic pain, opioids, primary care, continuing professional development, pain assessment, pain management, qualitative research, interdisciplinary team

## Abstract

**Background:**

Chronic pain (CP) is a debilitating disease that reduces quality of life, decreases productivity, and has become a primary cause of health care resource consumption. Despite this, many Canadian family physicians have received little formal education in managing CP, making it one of the most challenging areas of practice in primary care. Project Extension for Community Healthcare Outcomes Chronic Pain & Opioid Stewardship St. Joseph’s Care Group (Project ECHO-SJCG) is an evidence-based educational program connecting community-based health care providers (HCPs) with an interprofessional team by videoconference to learn about management of CP in rural, remote, and underserved areas.

**Aims:**

To explore key learning points from cases presented at Project ECHO-SJCG, identify and analyze themes and improve future sessions of continuing professional development for HCPs.

**Methods:**

We completed a thematic analysis of forty cases and key learning points using the constant comparison method. We also summarized descriptive statistics for patient and provider characteristics.

**Results:**

Forty cases were presented by 31 HCPs, who received suggestions focused on assessment and diagnosis, pharmacological and non-pharmacological pain symptom management, interventional management, attention to biopsychosocial factors, and appropriate referral to other HCPs.

**Conclusion:**

Project ECHO-SJCG cases allow HCPs to gain a broad knowledge base to evaluate and manage CP in their practice. Identified themes highlight common gaps in HCPs’ knowledge and will guide future sessions.

## RÉSUMÉ

**Contexte**: La douleur chronique est une maladie débilitante qui réduit la qualité de vie et diminue la productivité. En outre, elle est devenue une cause principale de consommation des ressources en soins de santé. Malgré cela, de nombreux médecins de famille canadiens ont reçu peu d'éducation conventionnelle sur la prise en charge de la douleur chronique, ce qui en fait l'un des domaines de pratique les plus difficiles en soins primaires.Le Projet de vulgarisation pour des résultats de santé communautaires – Gestion des opioïdes et de la douleur chronique du St. Joseph' s Care Group (projet ECHOSJCG) est un programme éducatif fondé sur les données probantes qui met les prestataires de soins de santé communautaires en relation avec une équipe interprofessionnelle par vidéoconférence pour en apprendre davantage sur la prise en charge de la douleur chronique dans les zones rurales, éloignées et mal desservies.**Objectifs**: Explorer les principaux points d'apprentissage à partir des cas présentés au projet ECHO-SJCG, recenser et analyser les thèmes et améliorer les futures sessions de développement professionnel continu pour les professionnels de la santé.**Méthodes**: Nous avons effectué une analyse thématique de quarante cas et points d'apprentissage clés à l'aide de la méthode de comparaison constante. Nous avons également résumé les statistiques descriptives pour le patient et le prestataire.**Résultats**: Quarante cas ont été présentés par 31 professionnels de la santé ayant reçu des suggestions axées sur l'évaluation et le diagnostic, la prise en charge des symptômes de douleur pharmacologique et non pharmacologique, la prise en charge interventionnelle, l'attention aux facteurs biopsychosociaux et l'orientation appropriée vers d'autres professionnels de la santé.**Conclusions**: Les cas du projet ECHO-SJCG permettent aux professionnels de la santé d'acquérir une large base de connaissances pour l’évaluation et la prise en charge de la douleur chronique dans leur pratique. Les thèmes recensés mettent en évidence les lacunes communes dans les connaissances des professionnels de la santé et orienteront les sessions futures.

## Introduction

Chronic pain (CP) is a debilitating disease. It interferes with people’s ability to work, sleep, and perform activities of daily life,^1–3^ leading to reduced quality of life. Affecting 1 in 5 Canadians,^4–6^ it is the primary cause of health care resource consumption and disability among working age adults.^7^ Despite its impact, health care providers (HCP) have historically received little formal education in management of CP,^8–15^ making it one of the most challenging areas of practice, especially in the context of the current opioid crisis.^16^

Opioids remain a common and important treatment option for the management of CP despite modest benefits in the management of CP, and association with considerable risks such as opioid misuse, overdose and death.^16–21^ Recent opioid prescribing guidelines and a fear of disciplinary action have led many HCPs to initiate opioid tapering or rotation for some of their patients with CP.^8,22^ Additionally, individuals who are suitable candidates for opioid therapy are having more difficulty obtaining prescriptions, as HCPs are worried about contributing to opioid diversion or misuse.^23–27^ This has led to increasing numbers of patients who seek opioids from illicit sources due to suboptimal pain control, as well as unintentional opioid-related deaths.^23,25–31^ HCPs need to have access to a range of educational opportunities that reflect the complexity of CP to address gaps in knowledge or skills they commonly identify.

Project Extension for Community Healthcare Outcomes (ECHO)^32^ is a continuing medical education program that connects HCPs to an interprofessional team (ECHO Hub) via videoconference to provide case-based discussion and didactic lectures. Since 2003, Project ECHO has been developed and reproduced across a variety of diseases and specialties around the world. A systematic review of 39 studies that reported on a median of 38 HCPs per study investigated the effects of ECHO on HCPs and patients across 17 illnesses (including CP). Four studies indicated that ECHO increased knowledge (3 studies evaluating perceived knowledge and 1 evaluating objective knowledge), eight studies reported improvement in self-reported competence, one study reported an increase in health-care access, and two studies reported various indicators of cost-effectiveness.^33^ Project ECHO has been applied to CP, with demonstrated improvements in self-efficacy and knowledge.^1,34^

Funded by the Ontario Ministry of Health, Project ECHO Chronic Pain and Opioid Stewardship St. Joseph’s Care Group (Project ECHO-SJCG) is composed of HCPs with experience working with CP populations and in remote, rural, and underserved areas. In addition to the minimum evaluation requirement for the Ontario Ministry of Health, our team is engaged in ongoing evaluation and quality improvement initiatives to guide program development and improve the overall educational value of Project ECHO-SJCG. As part of an evaluation of the program, we have undertaken a detailed analysis of the cases presented during the first year of Project ECHO-SJCG sessions. The goal was to describe the demographic and medical characteristics of patients that were the focus of case discussions and to derive common themes from the case discussions. The results will inform future Project ECHO-SJCG sessions (e.g., Hub membership, topics for didactic sessions, resources to be developed or curated for participants).

## Methods

### Design

We used a descriptive approach to summarize patient demographic and medical characteristics from the case report forms submitted by HCPs and we conducted a thematic analysis^35^ of forty case conference summaries from ECHO-SJCG sessions from May 2018 to March 2019.

### Setting

Project ECHO-SJCG’s Hub consists of specialists with expertise in the management of CP who are based at St. Joseph’s Care Group in Thunder Bay (Ontario, Canada), and at The Ottawa Hospital in Ottawa (Ontario, Canada). The Hub offers weekly 90-minute sessions to HCPs who are interested in learning about managing CP in their practice and includes a didactic presentation as well as a 45-minute case discussion. Cases are submitted by participants in advance by completing a detailed form with non-identifying information about their patient (case report form). During the case discussions, both participants and Hub members are invited to ask questions and offer their perspective on the case presented. A Hub member records the key learning points about the case on a case summary form which is subsequently (within 1 week) reviewed by all members of the Hub to ensure that it accurately reflects what was discussed before being shared with the presenting HCP and stored on the Hub website. Because Project ECHO-SJCG case discussions are not clinical consultations, we use the term “key learning points” to refer to the key ideas that are recorded about the case discussions.

### Epistemological Stance

Pain is a subjective phenomenon highly influenced not only by biological processes, but by psychological, social and in some cases spiritual factors that dynamically influence each other.^36,37^ Pain can never be purely objectively described and treated without using some form of language (verbal or non-verbal) which in turn can also impact its quality and intensity, and the outcomes of various pain management strategies. As such, the team approaches the field of pain and its management, and consequently this evaluation of Project ECHO Chronic Pain and Opioid Stewardship, from a constructivist stance.^38^

### Procedure

Case report forms and case summary forms were retrieved from the public registry available through Project ECHO Chronic Pain and Opioid Stewardship at St. Joseph Case Group for quantitative and qualitative analyses as described below.

### Quantitative Analyses

Two members of the research team (RC and AI) independently extracted HCP patient characteristics from the case report forms. The data was compared, and discrepancies resolved by returning to the data source.

### Qualitative Analyses

Analysis of qualitative data followed Braun and Clarke’s recommendations for the thematic analysis approach,^35^ using the qualitative analysis software NVivo12. Two members of the research team (IA, AG) independently familiarized themselves with the case summaries and key learning points through repeated reading of the material, and created preliminary coding categories inductively, using the constant comparison method. Members broke down each unit of meaning, assigned a code, and compared each unit of meaning to previously coded units to see if they represented the same idea. The reviewers met to review and discuss coding and create a codebook before coding all data available. We also held consensus meetings to discuss coding uncertainties through dialogue and at times through returning to the data source to better appreciate the context of a particular code. We also met to identify common and recurring themes, and discuss implications for the future program improvement (IA, AG, YS, PP).

## Results

### Provider and Patient Demographics

**Figure 1.**
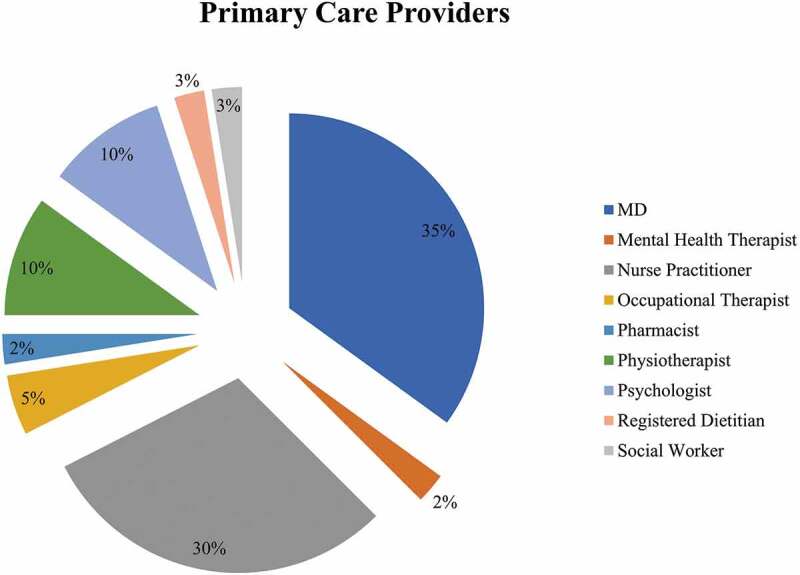
Percentage of health care providers represented by different health disciplines (n = 40).

**Figure 2.**
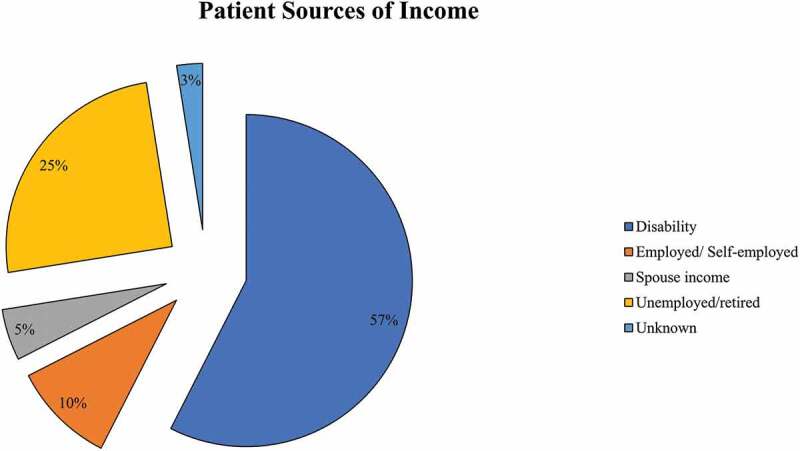
Patient sources of income (n = 40).

Forty cases were presented by 31 different HCPs, the majority of whom were family physicians and nurse practitioners (65%; see Fig. 1). Patients (52.5% males) presented were on average 54.6 years old (SD = 18.1). The majority (87.5%) were unemployed (see Fig. 2). Back (27.5%), extremities (22.5%) and head/neck (17.5%) were the most common pain locations reported. Fifty percent of patients were using short-acting opioids and 30% were using long-acting opioids to manage their pain. Common co-occurring medical problems were hypertension (45%) and asthma or chronic obstructive pulmonary disease (35%). Depression (40%) and anxiety (30%) were the most common mental health problems reported. Substance use was prominent including alcohol (32.5%), nicotine (32.5%), cannabis (27.5%) and illicit opioids (22.5%). With regards to previous treatments, 20% of patients had undergone surgery to address their pain and 7.5% had received epidural injections. The most common non-pharmacological (physical and psychological) approaches previously tried were physiotherapy (55%), occupational therapy (32.5%), and exercise (32.5%). See Table 1 for more details.

**Figure:**
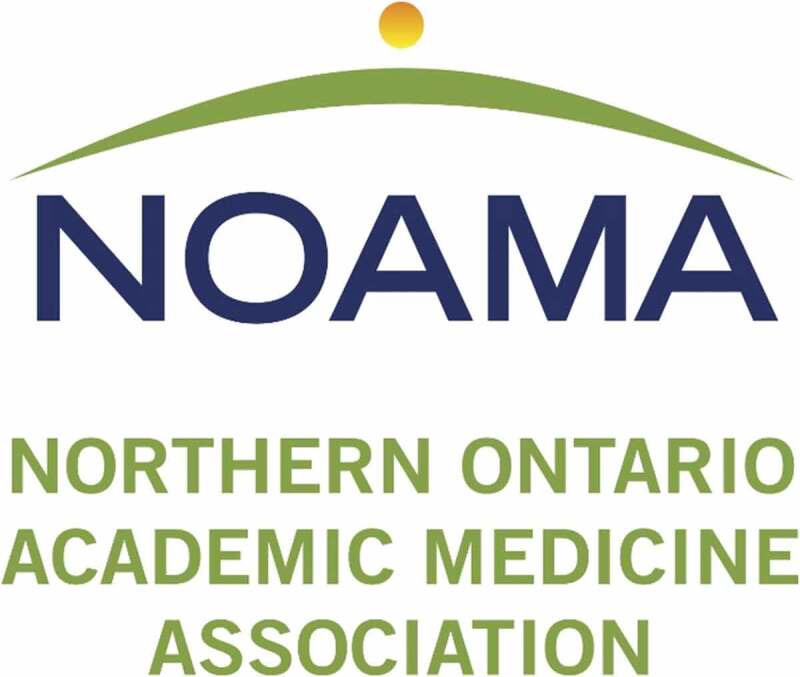

**Table 1.** Patient demographics, pain and medical characteristics.

				Total = 40
Age (years), Mean (SD)				54.6 (18.1)
**Sex**				**N (%)**
	Male			21 (52.5)
	Female			18 (45)
	Not specified			1 (2.5)
**Support System**				**N (%)**
	Family Support Present			32 (80)
	Social Support Present			32 (80)
	Formal Support Present			23 (57.5)
**Pain Locations**				**N (%)**
		Back		11 (27.5)
		Extremities		9 (22.5)
		Head/Neck		7 (17.5)
		Abdomen		5 (12.5)
		Joints		4 (10)
		≥3 locations		31 (77.5)
**Opioid Use**				**N (%)**
	Long Acting Opioids			12 (30)
	Short Acting Opioids			20 (50)
**Most Common Comorbidities**				**N (%)**
	Hypertension			18 (45)
	Asthma/COPD			14(35)
	Hyperlipidemia			11 (27.5)
	Sleep disorders			10 (25)
	Arthritis			9 (22.5)
	Diabetes			8 (20)
	Obstructive sleep apnea			5 (12.5)
	Peripheral vascular disease			4 (10)
	≥3 co-morbidities			12 (30)
**Associated Mental Health Conditions**				**N (%)**
	Depressive disorder			16 (40)
	Anxiety disorder			12 (30)
	PTSD			6 (15)
			Substance dependence	3 (7.5)
			Bipolar disorder	2 (5)
**Most Common Substance Use**				**N (%)**
			Alcohol	13 (32.5)
			Nicotine	13 (32.5)
			Cannabis	11 (27.5)
			Illicit opiate(s)	9 (22.5)
			Caffeine	7 (17.5)
**Treatments Previously Trialed**				**N (%)**
			Other	13 (32.5)
			Surgery	8 (20)
			Epidural injection	3 (7.5)
			Trigger point injection	2 (5)
			Joint injection	2 (5)
**Most Common Physical and Psychological Interventions Previously Trialed**				**N (%)**
			Physiotherapy	22 (55)
			Occupational therapy	13 (32.5)
			Exercise	13 (32.5)
			Self-management strategies (self-led, peer-led, and online)	10 (25)
			Cognitive behavioral therapy/counseling	10 (25)
			Mindfulness	9 (22.5)
			Relaxation strategies	9 (22.5)

### Thematic Analysis of the Key Learning Points

Thematic analysis of the case key learning points revealed six major themes: 1) Assessment and diagnosis; 2) Pharmacological pain and symptom management (including opioid prescription and management); 3) Non-pharmacological pain and symptom management; 4) Interventional management; 5) Attention to psychosocial factors; 6) Referral to other HCPs. For a summary of themes, sub-themes and key learning points please see Supplemental Table 1.

### Assessment and Diagnosis

The case discussions commonly revealed the need for additional information to identify the etiology of the patients’ pain problem, and guide management and treatment. Four sub-themes were identified: physical examination, imaging, laboratory testing, and differential diagnoses (see Table 2.Vignette 1).

**Table 2.** Vignette 1: “Assessment and diagnosis”.

Case information provided	This patient is a woman between the age of 55 and 65 who is looking for different ways to increase her stamina and physical activity. The provider goal was to explore alternative ways of managing pain related to fibromyalgia, which the patient had been previously diagnosed with. She also has osteoarthritis, stomach issues, acid reflux, occasional migraine headaches and diffuse body aches, and flu like symptoms. Furthermore, she had been diagnosed with bipolar disorder in the late 1990s (not currently medically treated) and her depression worsens when pain worsens (currently on Zoloft). Current medication list included: Pregabalin, Acetaminophen-Codeine, Lorazepam, and Sertraline Hydrochloride.
Lessons learned from the case	The community and hub suggested assessing for hypermobility (Beighton score and history), checking for inflammatory markers and a metabolic workup for rheumatological diagnoses, imaging (×-ray) of the cervical spine to assess for the possibility of severe degeneration given the osteoarthritis. The bipolar disorder diagnosis was also queried, given symptoms described, which would have implications for mental health support and treatment. Medical management options explored included rotating short acting opioids to a longer acting formulation, a slow taper of Lorazepam and a trial of Nortriptyline or Desipramine. Dietary suggestions included considering a trial of omega 3’s and fish oil DHA/EPA supplement. The team also suggested the development of a personalized fitness/exercise program, review of pacing and energy conservation techniques, development of a pain flare plan, along with strategies to manage anxiety and improve sleep (e.g., mindfulness, sleep hygiene).

Physical assessment suggestions focused on providing rationale for using specific tests along with detailed procedures and demonstration. This included, for example, a review of pertinent neurological and myofascial assessments for spasticity, assessing hypermobility, recognizing important presentations of co-occurring or causal conditions (e.g., examining the scalp, skin and nails for changes consistent with psoriasis), along with quantitative sensory testing exam for a more precise assessment of neuropathic pain and central sensitization.

Imaging teaching points included suggestions of specific diagnostic tests and their indications, along with discussions of less invasive and costly options when appropriate, such as x-ray or ultrasound in lieu of computerized tomography or magnetic resonance imaging. Laboratory testing was also discussed to assess potential metabolic imbalances, endocrine diseases, systemic inflammatory conditions, and chronic infective processes (e.g., hepatitis B/C or HIV). Where applicable, guidelines for urine drug testing for opioid stewardship were also discussed, along with their limitations (e.g., false positives).

Lastly, a variety of differential and potential co-occurring diagnoses were discussed. HCPs were encouraged to develop and apply a systematic approach that considers mechanical, inflammatory, physiologic, or infectious disease processes to achieve accurate diagnosis (e.g., myelopathy due to spinal stenosis or degenerative disc disease/osteoarthritis; seronegative spondyloarthropathy or postoperative abdominal myofascial wall pain; central sensitization; Lyme disease). HCPs were also encouraged to pay attention to psychological factors or mental health problems that can contribute to pain and impact functioning. A variety of assessment tools to aid in the consistent evaluation of patients’ status were also discussed (e.g., cognition, suicidality and hopelessness, opioid use disorder).

### Pharmacological Management

Four sub-themes were identified around pharmacological key learning points: trialing, titrating, or substituting medications, opioid prescribing and management, use of topical medications, and managing medication side-effects (see Table 3. Vignette 2). The first subtheme included discussions surrounding drug substitutions (e.g., selective norepinephrine reuptake inhibitor antidepressants and tricyclic antidepressants for neuropathic pain; beta-blockers for headaches), titration and modifications of administration schedule (e.g., dividing buprenorphine/naloxone doses to obtain more sustained analgesic effects), as well as the addition of co-analgesics (e.g., antidepressants for neuropathic pain). Off-label use of medications was also discussed in cases where other medications failed to produce adequate relief (e.g., alpha-blockers, beta-blockers, calcium-channel blockers). There was discussion of variability in patients’ responses and the need for a systematic approach in trialing different drugs.

**Table 3.** Vignette 2: “Pharmacological management”.

Case information provided	This patient is a woman between the ages of 36 and 45 who wants to be ‘pain free.’ The provider goal was to explore ideas for treatment and long-term management. The patient was diagnosed with clinoidal meningioma after a bout of severe orbital and temporal lobe pain. Initially, she had a right-sided headache only thought to be related to irritation of the trigeminal and facial nerves next to a cavernous sinus tumor. After the tumor was removed, pain became more generalized. She had a recurrence associated with worsening pain. She underwent MRI gamma knife procedure and completed radiation therapy. Her pain improved but she continued to use codeine, up to as many as one thousand tablets acetaminophen-codeine per month. She was then started on oxycodone acetaminophen and subsequently diagnosed with an opioid use disorder. She was partially weaned from her oxycodone acetaminophen, and she turned to acetaminophen-codeine. She is now taking three oxycodone acetaminophens per day and acetaminophen-codeine throughout the day.
Lessons learned from the case	After discussing a potential diagnosis of medication over-use headache (opioid and acetaminophen) and considering the opioid taper underway and its impact, the community and hub suggested pausing the opioid wean to focus on other aspects of the patient’s care. This included considering other prophylactic headache medications; nortriptyline is an option that would also help with sleep; a switch from pregabalin to Topiramate could be considered given that it is less likely to cause weight gain, but must be watched for cognitive side effects (start at a half-dose by sprinkling half of capsule contents over food); any tricyclic antidepressant could be taken earlier in the evening to reduce morning sedation; propranolol may also be helpful (also good for anxiety/agitation), but monitor for dizziness when standing. Dietary supplements suggested included magnesium to address constipation and pain. The team recommended an exploration of the patient’s functional goals and the use of motivational interviewing to better match interventions to the patient’s readiness to change. A referral to a brain injury day program for a more comprehensive and coordinated approach to physical, cognitive, and social difficulties. Lastly, exploration of options for income replacement (e.g., Ontario Disability Support Program) were suggested.

In the sub-theme of opioid prescription and management we highlighted HCP recommendations for safe prescribing practices which included discussing parameters for opioid prescribing (e.g., having one prescriber, discussing any changes with the HCP before making a change, attending appointments, secure storage), understanding side effects and potential drug interactions, using an opioid risk tool to assess potential for risk of opioid misuse and substance use disorder, suggesting the provision of a naloxone kit in case of overdose, along with monitoring of opioid therapy (i.e., pill count, urine drug screening, weekly dispensing) and opioid side-effects (e.g., bone loss, impotence).

Opioid rotations were also often described; a rotation to buprenorphine/naloxone due to a failed opioid trial, and transition from a short-acting to longer-acting opioid such as buprenorphine, were reviewed as management options. Additionally, when opioid tapering was suggested, it was recommended to be done gradually (e.g., 10% every 1 to 2 weeks), with attention to the potential role of co-analgesics. This was the case for a patient on high-dose opioids for CP, who had already discontinued use of Oxycocet and Percocet but who was still taking more than 90 mg of morphine equivalent daily with little effect on pain and function.

The role of topical analgesics was reviewed. Topical options explored included local anesthetics (e.g., lidocaine), compounded cream containing a combination of drugs (e.g., a cream with lidocaine, amitriptyline, and ketamine) as well as anti-inflammatory agents (e.g., diclofenac). Finally, considerable attention was given to the management of side effects from prescription drugs. This included the importance of reviewing medications to identify the potentially responsible agent causing unwanted effects (e.g., nabilone causing vertigo), consideration of a taper, use of an antagonist (e.g., Naloxogel for constipation due to opioid treatment) or discontinuation (e.g., in the case of headaches caused by antidepressants like SSRIs). In addition, education was provided on the importance of reducing polypharmacy (e.g., concurrent marijuana, benzodiazepine or opioid use affecting motivation to engage with other treatments), changing to another medication with fewer side effects, or adjusting the administration schedule (e.g., pregabalin taken at bedtime to reduce daytime sedation).

### Non-Pharmacological Management

With regards to non-pharmacological management, we identified three sub-themes: physical therapy, psychological therapy, and use of assistive devices. Many options for physical therapy were explored, with a predominant focus on maintaining and improving patient function, which could offer concurrent pain relief. These included hydrotherapy, graded motor imagery, mirror box and tactile discrimination exercises, as well as various mindful approaches to movement including yoga and Pilates. In some cases, this also included discussion of strategies to improve posture or positioning (e.g., ergonomic assessment of a workstation, modification of sleep position, use of pillows and cushions for support).

Psychological therapy focused on direct interventions that may require additional training or delivery by mental health professionals included psychological approaches focused on the management of pain (e.g., cognitive-behavioral therapy), mindfulness-based interventions, or psychological therapies to address co-occurring mental health needs (e.g., dialectical behavior therapy to address emotional dysregulation associated with complex trauma). In some cases, the use of motivational interviewing techniques was also recommended to support the client in clarifying their goals and values, and enhancing readiness for change. HCPs were encouraged to facilitate referrals to professionals within their communities with specialized training in specific psychological interventions (i.e., cognitive behavioral therapy, dialectical behavior therapy, mindfulness, and motivational interviewing). Assistive devices were also often discussed as strategies to increase function and subsequent pain control. This ranged from an assessment for custom ankle-foot orthoses (AFO) or braces, to recommending the use of a gait aid or light compression sleeves, or providing enlarging grips and handles to reduce strain on hands.

### Interventional Management

In the interventional theme we described discussions surrounding interventions with short-term and longer-term effects, along with conditions necessary to achieve maximal benefit. Injections were suggested for short-term use (e.g., trigger point injections, dry needling, injection of steroids for joint pain), with attention to the use of X-ray or ultrasound guidance to improve accuracy when necessary. Long-term strategies that were more invasive included radiofrequency ablations, spinal cord stimulation, and surgery.

### Psychosocial Factors

We identified four sub-themes relating to psychosocial factors: the patient/HCP relationship, education, goal setting and source of insurance coverage. Attention to the patient/HCP relationship was often a focus of discussion when mistrust from patients was identified by the case presenter. Ideas to improve the patient/HCP relationship included demonstrating care by open and transparent communication about efforts made to support the patient’s care such as discussing the fact that consultation with colleagues was sought and demonstrating thoroughness by making sure to investigate preexisting conditions. The importance of approaching a patient with complex needs with fresh eyes, as though they were a new patient, was also discussed as a strategy to support HCP in letting go of some pre-conceived ideas, which could be impacting the patient/HCP relationship.

Regarding education, there was an emphasis on HCPs assisting their patient in mitigating common challenges associated with living with pain. This included articulating or finding purpose, developing healthy interpersonal relationships, encouraging socialization, and engaging in self-management strategies to improve quality of life and function. Education about pain to reduce fear and hypervigilance and emphasizing the importance of nervous system health and mental health (e.g., role of anxiety and depression in CP) in pain management were common key learning points. Additionally, strategies to communicate effectively with patients about the importance of managing co-occurring conditions which may contribute to pain, such as diabetes, were discussed (e.g., motivational interviewing).

Finally, goal setting was commonly explored with presenters using the SMART (specific, measurable, attainable, realistic, time-bound) strategy; for example, dividing a large goal into small feasible steps to increase motivation, and developing action plans to increase fitness levels or improve day-to-day functioning. Lastly, there were frequent discussions about paying attention to patients’ insurance coverage when recommending treatments (e.g., Non-Insured Health Benefits for First Nations and Inuit, Ontario Disability Support Program, private health insurance); noting that different medications and treatments are covered by different insurance providers. HCPs were encouraged to consider this to ensure that the treatments they recommend are accessible.

### Referral to Other HCPs

The last theme of referrals was grouped into three subthemes: suggestions for extended care in primary community-based settings, referrals to specialist care, and referrals for interprofessional/group-based programs. Primary or community-based care referrals focused on being aware of the services offered in the community, including physical/manual therapy, occupational therapy, psychologist, social work, substance use care, or a family physician with a focused practice.

Specialist care referrals were frequently discussed, especially when additional assessment was required or when the HCP felt the patient’s needs were complex and transcended their level of knowledge or skillset. This included specialists or specialty assessment clinics with the intention of confirming diagnosis, optimizing disease control, and/or managing comorbid conditions (e.g., rapid access clinics for hip/knee arthritis, polysomnography for sleep disorders, psychiatry for the management of co-occurring mental health problems, podiatrist for gait analysis and correction, rheumatology for assessment of auto-immune disorder).

Lastly, participation in interprofessional pain management programs was often discussed as a holistic approach to pain management. Community-based health professional-led or peer-led groups were commonly recommended to assist patients in acquiring specific skills (e.g., dialectical behavior therapy skills group) or to sustain engagement in physical activity (e.g., online conditioning programs).

## Discussion

Cases brought forward for discussion were complex, and demonstrated the common interplay among CP, mental health and substance use that HCPs face in clinical practice. We found that 50% of patients presented during case discussions reported opioid use; in contrast about 17.6% of people living in Canada are prescribed long-term opioid therapy.^36^ Rates of depression and anxiety reported were similar to previous reports in the literature.^39,40^ People with more complex presentations of CP, including co-occurring mental health or substance use problems, face additional barriers to receiving^41^ and accessing care,^42^ despite recognition that management of mental health (e.g., depression) and substance use concerns^43^ are necessary for people to achieve significant improvements in pain and functioning.^44^ Project ECHO provides an opportunity for HCPs to learn about different treatment and management options.

The themes we identified highlight that Project ECHO-SJCG provides broad continuing professional development around key areas of CP management, including assessment and diagnosis, pharmacological and non-pharmacological management, attention to biopsychosocial factors contributing to pain, overall functioning, access to treatment, interventional management, and referral to other HCPs. Though ECHO-SJCG sessions are open to all HCPs, most of the presenting participants were physicians and nurse practitioners. Although this could have led to a narrow focus on medical management, non-medical management was the focus of 40% of key learning points, demonstrating the diversity of perspectives presented during sessions.

A review of case report forms and key learning points from the case-based discussions revealed a lack of information to support a clear diagnosis and management plan. This was sometimes because the HCPs had only known the patient for very short time. On other occasions, the need for further information arose from discussion of a differential diagnostic not previously considered. Other studies of Project ECHO case reports have observed that further physical examinations (e.g., checking for a nerve entrapment) and assessment of co-occurring conditions is often needed.^45–47^ Similarly, another concern was the of lack of details about specific treatment protocols that had been tried before (e.g., blanket statement about physiotherapy having been tried versus reference to a specific physiotherapy protocol). This requires broad knowledge about different approaches that may be indicated for different conditions, which is one of the goals of Project ECHO-SJCG.

One of the advantages of case-based approach to medical education is deeper learning.^48^ It may be possible to improve the Project ECHO-SJCG through having a few index cases to follow longitudinally over a few months, offering opportunities for ongoing dialogue at ECHO sessions as new information emerges from case presenter’s continued contact with patients. This would allow HCPs to conduct the necessary assessment and testing, have more in-depth discussions with their patients about prior treatment trialed, and have time to apply new knowledge and skills while being supported by Project ECHO-SJCG. An ongoing case discussion would also allow for an exploration of barriers and facilitators to HCPs’ behavior changes in the context of chronic disease management.^49^

Key learning points included considerable attention to the pharmacological management of pain, including the use of opioids. This may be because most presenting HCPs were physicians and nurse practitioners, and 50% of patients were prescribed opioids with 20% having a history of illicit opioid use. Opioids for CP remains a commonly used treatment, although it may only confer modest benefits with an increased risk of opioid use disorder and overdose.^8,9^ Although many options for learning about the role of opioids in the management of CP and strategies to trial and discontinue opioids exist, there is a clear need for and interest in synchronous discussions of patient cases. These discussions could be enhanced by applying existing tools to support clinicians in taking a comprehensive substance use history,^50^ strategies for shared decision making around opioid use for pain,^51^ and resources to support tapering and harm reduction.^52^

In contrast to opioid treatment, few patients presented had trialed interventional management, and this was addressed in Project ECHO-SJCG with discussion of when different procedures would be applicable. This may reflect the fact that interventional management may not be appropriate given pain diagnoses or co-occurring conditions, HCPs’ understanding of their applications and limitations, as well as lack of access to specialized centers where these are offered. Future Project ECHO-SJCG sessions could include didactic sessions and resources to assist HCPs in making decisions about best-practice interventional management, workshops offering training in the provision of some interventions (e.g., trigger point injections), as well as strategies to assist patients in accessing these services when indicated to achieve greater health care equity. For example, using Ontario eConsult (an electronic consultation platform for HCPs to connect with specialists)^47^ to confirm the applicability of interventional management and ensure continuity of care for follow-ups may improve the patient experience and increase health system efficiency. Project ECHO-SJCG sessions could include an orientation to eConsult as well as connection with health system navigators to augment the value for patients and providers.

Project ECHO-SJCG case discussions spanned the breadth of the biopsychosocial framework. In the cases presented, more than half of the patients had received physical rehabilitation (55%), which can target underlying pathology, improve functionality, and limits invasive procedures.^53^ However, despite the well-established role of psychological and social factors in the development, maintenance and management of CP, only 25% of patients from our sample had ever tried cognitive behavioral therapy, counseling, mindfulness training, or been taught relaxation strategies.^54–57^ This may reflect HCPs’ lack of training in the application of these strategies, lack of time during consultations to explore psychological therapeutic targets, as well as lack of access to these treatment modalities in the communities where patients live.^58–60^ Future Project ECHO-SJCG sessions could provide suggestions to HCPs for accessing training in the application of psychologically-based interventions, with attention to strategies that can be readily integrated in their practice (e.g., motivational interviewing) while maintaining an awareness of the boundaries of their scope of practice.

The advent of many online programs that can be self-directed or delivered with coaching support from HCPs present opportunities to further integrate psychological treatment modalities in the management of CP.^61–69^ Future Project ECHO-SJCG sessions could provide an orientation to these programs and support HCPs to connect with program facilitators to integrate these programs into their practice and encourage treatment of mental health conditions.^47^

Social factors were acknowledged during case presentations, but few key learning points addressed underlying social contributors to distress and pain such as isolation, trauma, family dynamics and poverty.^70^ These factors increase the complexity of case discussions, may appear to be outside of the direct scope of practice for presenting HCPs, and are typically more challenging to address than concrete therapeutic options that can be trialed. Strategies to improve Project ECHO-SJCG impact in this regard may include: 1) systematically acknowledging these factors during case discussions; and 2) having a systems navigator ready to provide resources to support HCPs in addressing these factors with their patients.

This study highlighted that attention is paid to strengthening the patient-HCP relationship during Project SJCG-ECHO, but this could be enhanced. Patients are more likely to adhere to treatment recommendations and realize benefits when a trusting therapeutic relationship exists between the HCP and patient.^71,72^ HCPs are often hesitant to engage patients in conversations about opioid harms, tapering and monitoring, due to concern over rupturing the patient-HCP alliance.^73–76^ We noted that many of the key learning points about the patient-HCP relationship were task-oriented (e.g., demonstrating care by indicating to patients that consultation was sought) rather than relationship-oriented, such as identifying and repairing ruptures,^77^ validation of patients’ subjective pain experience,^78^ ensuring accurate empathy,^79^ and maintaining unconditional positive regard.^80^ Project ECHO-SJCG could further enhance its curriculum by including didactic sessions focusing directly on these critical elements of the patient-HCP relationship.^81–83^ Furthermore, given the importance of education and self-management strategies in CP, HCP sessions could focus on strategies to: reduce fear of pain; facilitate goal setting with self-monitoring; and enhance social support and healthy interpersonal relationships.^84–89^

ECHO-SJCG aims to educate HCPs so the management of CP is improved within the context of primary care, and unnecessary referrals are minimized. While ECHO-SJCG’s work may obviate the need for further referrals, it is just as likely to improve HCPs’ referral practices, their ability to navigate complex medical systems, and assist patients in accessing resources. This includes accessing low cost, online and peer-led self-management programs, and exploring services that are offered within the community or nearby communities that presenters were not aware of. This points to the importance of the health care navigation role, which is often done by social workers or other well-connected health professionals but could also be embedded within Project ECHO-SJCG to support HCPs, particularly those working in remote, rural, and underserviced areas.^90^

Our findings are in line with those of other studies examining support mechanisms for HCPs. For example, in a study examining the HCPs’ use of a secure consultation platform to consult pain specialists, common themes identified included pharmacological management and safe opioid prescribing, the importance of addressing biopsychosocial factors, and encouraging participation in self-management programs.^47^ Carlin et al. reported five main themes they derived from focus group discussions on Project ECHO participants’ experiences and assessment of ECHO. These themes included 1) challenge of managing CP in primary care; 2) improvement in patient-HCP interaction and knowledge uptake; 3) sharing of the knowledge gained from ECHO with participants’ colleagues and patients; 4) creating a community through Project ECHO; and 5) drawbacks of participating in ECHO.^91^

A limitation of this study is that the thematic analyses focused on the written summaries provided in the case report forms and key learning points at each session. Although these case report forms were reviewed for completeness and accuracy by all members of the Project ECHO-SJCG Hub, it is possible that they were incomplete. The inclusion of a medical stenographer during case discussions would provide richer documentation for analysis but would also be more costly. We did not collect pertinent information about the types of patients presented by the HCPs during the ECHO discussion series. Insufficient patient information precludes us from commenting on the types of patient presentations that HCPs find challenging to manage. Furthermore, as this project focused on the characteristics of cases presented and key learning points, it did not assess whether participation in ECHO led to HCPs’ behavior change and improved patient outcomes. The current study focused on insights to be drawn from the type of cases and key learning points of Project ECHO-SJCG.

## Conclusion

Project ECHO-SJCG provides a comprehensive applied curriculum spanning the assessment, diagnosis, treatment, and management of CP. One strategy to improve Project ECHO-SJCG, which may be useful for other ECHOs, is to integrate index cases followed longitudinally over a period of several months, allowing for deeper engagement and attention to facilitators and barriers to HCPs implementing the knowledge and skills they learn through the sessions. Greater attention to strategies to improve patient-HCP relationships, such as the integration of a systems navigator in the Hub, is an additional key insight to improve the value of Project ECHO-SJCG for participants.
